# Homing and reparative effect of intra-articular injection of autologus mesenchymal stem cells in osteoarthritic animal model

**DOI:** 10.1186/1471-2474-12-259

**Published:** 2011-11-15

**Authors:** Abir N Mokbel, Omar S El Tookhy, Ashraf A Shamaa, Laila A Rashed, Dina Sabry, Abeer M El Sayed

**Affiliations:** 1 Department of rheumatology and rehabilitation, Faculty of Medicine. Cairo University, Egypt; 2 Department of medical biochemistry and molecular biology, Faculty of Medicine. Cairo University, Egypt; 3 Department of surgery, anesthesiology and radiology, Faculty of Veterinary Medicine. Cairo University, Egypt; 4 Department of pathology, National Cancer Institute. Cairo University, Egypt

**Keywords:** MSCs, Chondral defect, Intra-articular, Homing, GFP, Repair, Donkeys

## Abstract

**Background:**

This work aimed to study the homing evidence and the reparative effect of mesenchymal stem cells (MSCs) in the healing process of induced osteoarthritis in experimental animal model (donkeys).

**Methods:**

Twenty-seven donkeys were equally divided into 3 groups based on the observation period after induction of arthritis (3, 6 and 9 weeks) to achieve different degrees of osteoarthritis. Each group was subdivided into three subgroups of three animals each based on the follow-up period (1, 2 and 6 months) after treatment. The induction was done through intra-articular (IA) injection of 2 ml of Amphotericin-B in both carpal joints. MSCs were harvested in a separate procedure, labeled with green fluorescent protein (GFP) using monster GFP vector and suspended in hyaluronic acid for IA injection. Treatment approaches consisted of cell-treatment using MSCs suspended in 3 ml of hyaluronic acid (HA) for the right carpal joint; and using the same amount of (HA) but without MSCs for the left contralateral carpal joint to serve as a control. Animals were assessed clinically and radiologically before and after treatment. Synovial fluid was also evaluated. Histopathologically; articular cartilage structural changes, reduction of articular cartilage matrix staining, osteophyte formation, and subchondral bone plate thickening were graded. Data was summarized using median and percentile for scores of histopathologic grading. Comparison between groups was done using non-parametric Mann Whitney test.

**Results:**

The reparative effect of MSCs was significant both clinically and radiologically in all treated groups (P < 0.05) compared to the control groups. Fluorescence microscopy of sections of the cell-treated joints of all animals indicated that the GFP-transduced injected cells have participated effectively in the reparative process of the damaged articular surface and have integrated within the existing articular cartilage. The cells were associated with the surface of the cartilage and, were also detected in the interior.

**Conclusions:**

Homing was confirmed by the incorporation of injected GFP-labeled MSCs within the repaired newly formed cartilage. Significant recovery proves that the use of IA injection of autologous MSCs is a viable and a practical option for treating different degrees of osteoarthritis.

## Background

Adult marrow-derived Mesenchymal Stem Cells (MSCs) are capable of dividing and their progeny are further capable of differentiating into one of several mesenchymal phenotypes such as osteoblasts, chondrocytes, myocytes, marrow stromal cells, tendon-ligament fibroblasts, and adipocytes. In addition, these MSCs secrete a variety of cytokines and growth factors that have both paracrine and autocrine activities. These secreted bioactive factors suppress the local immune system, inhibit fibrosis (scar formation) and apoptosis, enhance angiogenesis, and stimulate mitosis and differentiation of tissue-intrinsic reparative or stem cells. These trophic effects are distinct from the direct differentiation of MSCs into repair tissue [1].

The use of MSCs for cell therapies relies on the capacity of these cells to home and engraft long-term into the appropriate target tissue [2]. MSC therapy has been applied in bone and cartilage repair and in the treatment of osteoarthritis [3].

Osteoarthritis (OA), the most common form of joint disease, is characterized by degeneration of the articular cartilage and, ultimately, joint destruction [4]. Loss of articular cartilage; caused by mechanical and oxidative stresses, aging or apoptotic chondrocytes; provoke synovial lining cells and articular chondrocytes within diseased cartilage to synthesize and secrete proteolytic enzymes, such as matrix metalloprotinases (MMP), aggrecanases, proinflammatory cytokines and mediators such as nitric oxide and prostaglandins which degrade the cartilaginous matrix [5,6].

Despite the high prevalence and morbidity of osteoarthritis (OA), an effective treatment is currently lacking. Restoration of the diseased articular cartilage in patients with OA is the challenge [4]. Difficulties in studying osteoarthritis in humans that stem from both the low sensitivity of diagnostic tools and the low availability of diseased tissues explain why research on animal models remains highly dynamic. Several animal models have been studied. Animal models of osteoarthritis (OA) include spontaneous models in aging animals, genetically modified mice, as well as surgically, enzymatically or chemically induced models [7]. IA injection of Amphotericin-B consistently resulted in aseptic arthritis in horses [8-14].

In clinical settings, the optimal route for administration of stem cells depends on the anatomy and the extent of damage of the involved tissue or organ, offering a choice between two approaches: direct local or intralesional implantation versus systemic intravascular administration. Site-directed delivery of MSCs has shown their engraftment in several tissues, particularly after injury. Several research work have discussed the use of bone marrow cells to repair infarcted myocardium [15,16], repair of spinal cord injuries [17-19] and in treatment of large cartilage defects [4]. As a result, cartilage repair with direct intra-articular injection (IA) of MSCs has been proposed as a potential cell therapy in a model of OA [20,21].

This work aimed to study the homing evidence and the reparative effect of intra-articularily injected mesenchymal stem cells (MSCs) in the healing process of experimentally-induced animal model (donkeys) of osteoarthritis having different degrees of osteoarthritis (mild, moderate and severe) and followed up for 1, 2 and 6 months after treatment.

## Methods

### Study design

**Figure 1:**
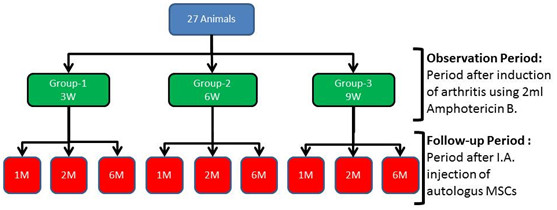
**Schematic diagram of the animal groups showing the induction of different degrees of arthritis with the following observation period, then the treatment stage with the follow up period**.

Induction of three different degrees of arthritis in twenty-seven animals using the same technique developed by [14]. Animals were equally divided into 3 groups (9 each) based on the observation period (3, 6 and 9 weeks). Each group was subdivided into three subgroups (3 animals each) based on the follow up period (1, 2 and 6 months) after cell-treatment (Figure 1). Bone marrow (BM) was harvested from each animals. MSCs were identified, labeled with green fluorescent protein (GFP) and suspended in hyaluronic acid for I.A. injection. Each animal received a single shot of autologous cell-treatment (MSCs suspended in Hyaluronic acid) in the right carpal joint. The left carpal joint served as a control and received hyaluronic acid only. Animals were assessed clinically; before and after treatment. Radiological, Synovial fluid analysis and histopathological assessment were performed.

#### 1- Preparation of experimental animal model and Induction of arthritis

This animal experiment followed the guidelines developed by the American Psychological Association (APA) for the ethical conduct of care and use of animals [22] and approval was obtained from the faculty of Veterinary medicine, Cairo University, Egypt. All animals were prepared in the same manner. Mild, moderate and severe degrees of arthritis were achieved 3, 6 and 9 weeks respectively after IA injection of 2 ml of Amphotericin-B 50 mg (Fungisone 50, 000 I.U.) in both carpal joints of each animal [14].

#### 2-Pre-treatment Follow-up

##### 2.1-Clinical Assessment

Evaluation of lameness was done following the American Association of Equine Practitioners (AAEP) scale for lameness evaluation [23]; (0 = Sound; 1 = Lameness difficult to detect and inconsistent; 2 = Lameness difficult to detect, but consistent; 3 = Lameness consistently detectable on a straight line; 4 = Obvious lameness with marked head nodding).

##### 2.2-X-Ray filming

X-ray films of the carpal joints were taken prior to any interference. The settings of the x-ray machine and the degree of arthritic changes were scored using a modified [9]. The score ranged from0 to 4 scale (Table 1). Radiographs were taken before induction (injection of amphotericin B), of each carpus for each animal, and on experimental days 0, and weekly afterwards.

**Table 1:** Crawford Radiographic scoring system

Radiographic evaluation
0 = normal
1 = no bone or cartilage change, slight joint distention and effusion.
2 = minimal bone changes, osteophytes < 1 mm, without evidence of cartilage loss
3 = moderate bone changes, osteophytes 1- 2 mm, bone lysis or cartilage loss
4 = severe bone changes, osteophytes > 2 mm, with/without evidence of cartilage loss and/or bone lysis

##### 2.3-Synovial Fluid

Synovial fluid was aspirated in a sterile syringe for analysis. Synovial analysis, included: physical properties (color, viscosity), Biochemical parameters: alkaline phosphatase (ALK), aspartate aminotransferase (AST), Alanine aminotransferase (ALT) Lactic acid dehydrogenase (LDH), and total proteins (TP) together with cytological analysis [14]. Animals were securely controlled, casted laterally. Two samples from each carpal joint were collected from each animal, immediately prior to MSCs injection (post-induction) and at the time of sacrifice (post-treatment).

#### 3- Acquisitions of Bone Marrow

Four weeks before any cell-treatment, bone marrow samples were taken from the lateral side of the proximal portion of the humerus bone, from the area below the head of the humerus and above the trochanter major. Animals were anaesthetized and securely controlled on lateral recumbency. The hair on the shoulder region was clipped, shaved and the area was disinfected with chlorohexidine then touched with Bovidone Iodine preparation. A bone marrow needle 14 G (2.0) was used to reach the bone marrow cavity after being moistened with Heparin Sterile syringe of was used to aspirate 20 cc of the bone marrow on 2 cc of 1500 IU of Heparin.

#### 4- Laboratory work: Preparation of mesenchymal stem cells

The lab work consisted of three major steps: Isolation; characterization; culturing and labeling of MSCs.

##### 4.1-Isolation of MSCs

Under complete aseptic technique; the isolation of MSCs was performed [24]. The bone marrow aspirate was diluted 1:3 with stromal medium consisting of DMEM-Ham's F12 medium (vol/vol, 1:1; HyClone, Logan, UT), layered onto Histopaque-1077 (Sigma, St Louis, MO, USA) and centrifuged at 400 g for 30 min. The collected buffy coat was mixed with 20 ml of Dulbecco's phosphate-buffered saline (DPBS) and centrifuged at 300 g for 5 min. The supernatant was discarded and the cells pellet was washed two more times with DPBS. After determination of cell viability and the number of viable cells by trypan blue staining, the washed pellet cells was re-suspended in DMEM-Ham's F12 medium (Sigma) supplemented with 10% fetal bovine serum (FBS; USDA, Gibco, Grand Island, NY, USA), antibiotics (penicillin 10 000 U⁄ ml, streptomycin 10 000 U ⁄ml) and Amphotericin-B 25 U⁄ml. This medium was also used as a control medium for the experiments. The nucleated cells were plated as primary culture in tissue culture flask at 2.5 × 10^5^⁄cm^2 ^and incubated at 37°C in a humidified atmosphere containing 5% CO_2_. On day 4 of culture, the non-adherent cells were removed along with the change of medium every 2 days. Undifferentiated MSCs were transplanted in this study upon reaching 70-80% confluence. The cells were counted with a hemocytometer and resuspended in 3 ml of hyaluronic acid at a final density of 1.8-2.3 × 10^6^cells/ml prior to intra-articular injection.

##### 4.2-In vitro Characterization of MSCs

Cells were identified as being MSCs by their morphology; the adherent colonies of spindle fibroblast like- cells were trypsinized, and counted. MSCs phenotypes were confirmed by flow cytometry and analysis of cell surface molecules as detailed elsewhere [25] for CD34^- ^and CD29^+^. Cells were sorted by using FITC-labeled anti-CD34 (1:20; DAKO, Carpinteria, CA, USA), and anti-CD29 (1:20; DAKO). Briefly, after staining with appropriately conjugated antibodies (Ab) and washings, cells were analyzed on a BDL cytofluorimeter, (BD Biosciences). The area of positivity was determined using an isotype matched control Ab. 10^4 ^events for each sample were acquired. They were also characterized by their in vitro power to differentiate into osteocytes and chondrocytes [26]. For osteogenic induction, MSCs were plated at 2 × 10^4 ^cells/cm^2 ^and cultured in osteogenic differentiation medium (DMEM supplemented with 10 mM β-glycerophosphate, 10-8 M dexamethasone, and 0.2 mM ascorbic acid) for up to 20 days, with medium changed three times per week. For chondrogenic induction, MSCs were pelleted and cultured in chondrogenic differentiation medium (DMEM supplemented with 0.1 μM dexamethasone, 0.17 mM ascorbic acid, 1.0 mM sodium pyruvate, and 0.01 μg/ml transforming growth factor-β (Peprotech, London) for 28 days with medium changed three times per week. The micromass pellets were formalin fixed, paraffin embedded, and sectioned in slices. Thereafter, in vitro differentiation into osteocytes and chondrocytes was confirmed by alizarin red and alcian blue stains for osteocytes and chondrocytes respectively in cells culture pellet.

##### 4.3-Labeling of MSCs

Undifferentiated MSCs were harvested and were labeled with green fluorescent protein (GFP) using monster green fluorescent protein vector and lipofectamintransfast transfection reagent kit (Promega, Madison, WI, USA). Before transfection 3 - 5 × 10^5 ^cells were seeded into individual wells of 6 well-plates. After 24 h incubation in growth medium, the cells were exposed to 2 μg GFP plasmid/well of cells. GFP plasmid was incubated with lipofectamin for 10-15 minutes before subjection to the cells. Following transfection the cells were incubated at 37°C in humidified air (5% CO_2_) for 2 h. The transfection medium was then removed and the cells were incubated for an additional 48 h in complete medium (2 ml per well) [27]. For imaging GFP auto-fluorescence of MSCs, unstained slides were directly analyzed by confocal laser microscopy (LSM 510, Zeiss, Jena, Germany) incorporating two lasers (Ar and HeNe) equipped with an inverted Axiovert 100 M microscope [28].

#### 5-Injection of MSCs

Based on the timetable provided (Additional file 1); immediately following the aspiration of the synovial fluid and at the same procedure, each animals received its designated autologous MSCs IA injection coupled with Hyaluronic acid on its right carpal joint, while the left carpal joint was injected with Hyaluronic acid only.

#### 6-Post-treatment follow-up

Clinical Assessment, X-Ray Filming and Synovial fluid sample analysis were done in the same manner as the pre-treatment assessment

#### 7- Sampling

At the end of the experiment, and according to the sacrifice table, euthanasia was done. The skin was removed from the carpi and transverse cuts were made with a band saw through the radius just above the distal epiphysis and through the metacarpal below the carpo-metacarpal joint. The carpal canal was removed to allow complete extension of the carpus. The carpus was then opened at radio-carpal joint, metacarpal joint. The synovial membrane and fibrous joint capsule could be examined. The cartilage surface of the bone, each cut, also, was carefully examined.

#### 8- Assessment of homing

For assessment of homing of MSCs, unstained paraffin-embedded 4 *μ* m thick sections were examined by fluorescent microscope for detection of GFP-labeled stem cells in the newly formed cartilage.

#### 9-Histopathologic and Histochemical Assessment

Histologic assessment of the articular surface of the radio carpal joints for all animals was done to determine if there were differences between the MSCs-treated and untreated groups. The pathologist was totally veiled from the sample numbers and groups of this study. All cases were fixed in 10% neutral buffer formalin. De-calcification of tissue cases were done by using 8% formic acid decalcifying solution in distilled water. The decalcifying solution was renewed every 48 hours until softening of the tissues. The decalcified specimens were then trimmed, washed and dehydrated in ascending grades of alcohol, cleared in xylene, embedded in paraffin, sectioned at 4-6 μm thickness and stained with haematoxylene and eosin as well as Masson's trichrome stain for detection of collagen fibers and degree of matrix staining. Alcian Blue-PAS stain was used for staining of the acidic glycosaminoglycans. The appearance of the blue color in the areas of cartilage with pathological morphology chemically indicates enrichment of acidic glycosaminoglycans. Histologically it indicates newly formed cartilaginous tissue [29].

#### 10-Semiquantitative Histological Scoring

Articular cartilage structure, reduction of articular cartilage matrix staining, changes in osteophyte formation, and subchondral bone plate thickening were graded according to [20] as described in table 2.

**Table 2:** Histopathologic grading system

Parameter & Grade	Description
**Articular cartilage structure**	
0 - 10 (30)	0 = normal, 10 = complete loss to subchondral bone
**Reduction of articular cartilage matrix staining**	
0	None
1	Mild
2	Moderate
3	Severe
**Presence of osteophytes**	
0	None
1	Cartilage \ connective tissue
2	Mainly cartilage\ some bone formation
3	Mainly bone formation
**Subchondral bone plate thickening**	
0	None
1	Mild
2	Moderate
3	Severe

#### 10-Statistical Analysis

Data was coded and entered using statistical package SPSS version 15. Data was summarized using median and percentile for scores of histopathologic grading. Comparison between groups was done using non parametric Mann Whitney test. P values less than or equal to 0.05 were considered as statistically significant.

## Results

### I- Mesenchymal stem cell identification and characterization

**Figure 2:**
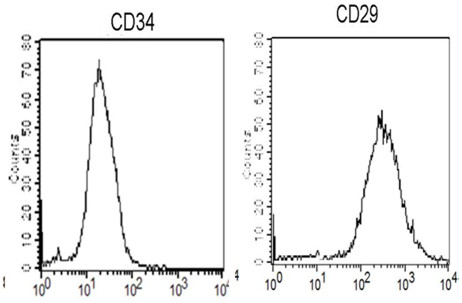
**Flow cytometric characterization analyses of bone marrow-derived MSCs**. Cells were uniformly negative for CD34, and positive for CD29.

MSC identification and characterization was done by the phenotypic analysis of the cells. Flow cytometric characterization analyses of bone marrow-derived MSCs showed that the cells were uniformly negative for CD34 and positive for CD29 (Figure 2).

**Figure 3:**
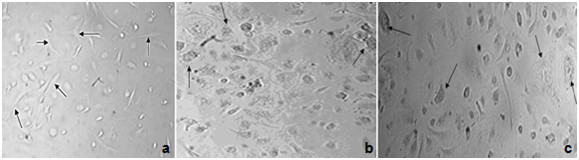
**(a) MSCs-BM cells in culture without adding growth factors for osteogenic and chondrogenic differentiation arrows show fibroblast-like cells in morphology**. (b) MSCs-BM cells in culture after adding growth factors for osteogenic and (c) chondrogenic differentiation arrows show change in MSCs morphology.

**Figure 4:**
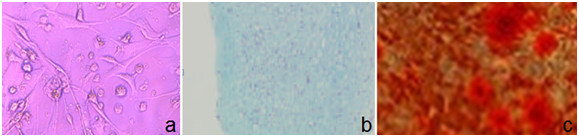
**(a) Control undifferentiated MSCs showed neither staining with Alzarin red (special stain for differentiated MSCs into osteoblasts) nor Alcian blue (special stain for differentiated MSCs into chondrocytes); (b) Osteogenic differentiation of these cells shows the presence of alizarin stained calcium deposits in MSCs-BM; (c) Chondrogenic differentiation of MSCs-BM shows the presence of Alcian blue stained of differentiated cells**. All these images are at a magnification of 20X.

Undifferentiated MSCs were identified in vitro by its characteristic adhesive morphology (fibroblast like cell) as labeled by arrows in Figure 3a. The ability of MSCs to differentiate into osteoblast and chondrocytes were identified in vitro by changing their morphology as labeled by arrows in Figure 3b, c and by their staining with special staining as Alzarin red and Alcian blue for differentiated cells respectively as shown (Figure 4).

### II-Assessment before and after intra-articular GFP-labeled MSCs

Cell-treated joints (right carpi) were assessed at pre- and post-treatment with MSCs in hyaluronic acid and compared with control joints (left carpi) treated only with hyaluronic acid. Assessment results were:

#### II-1-Clinical lameness and swelling

Clinical signs in the form of acute swelling and joint capsular distention began within the first 3 hours post induction. The enlargement of the joint circumference was evaluated to have an average increase of 1 ± 0.24 cm. In the control joint, lameness in group-I was difficult to detect and inconsistent (score 1) whereas in group-II, lameness was difficult to detect, but consistent (score 2). In group-III, lameness was obvious with marked head nodding (score 4). Joint stiffness detected through visual inspection and passive bending of the joint. Stiffness was mild at 1 month and moderate at 2 month follow-up periods. Marked joint stiffness was clearly felt on examination in the left joints at 6 month follow-up period in all groups.

After intra-articular injection of GFP-labeled MSCs, all animals in all groups showed no improvement in motion after 1 month of treatment. In **group-I; **improvement was seen in animals kept for 2 and 6 months (score 1 and 0 respectively). Similarly, animals in **group-II; **improvement was seen in animals lasted for 2 and 6 months (score 2 and 1 respectively). Animals in **group-III; **showed improvement in animals lasted for 2 and 6 months (score 4) as shown in the table 3.

**Table 3:** Mean values of the lameness scoring before and after treatment for all groups at 1, 2, 6 months from the treatment

Assessment time	Group-I		Group-II		Group-III	
**Before Treatment**	2		3		5	

	**R**	**L**	**R**	**L**	**R**	**L**

**1 month After Treatment**	2	2	3	3	5	5

**2 month After Treatment**	1	2	2	2	4	5

**6 month After Treatment**	0	1	1	2	4	4

**R**: right joint; **L**: left joint.

#### II-2. Radiological findings

**Figure 5:**
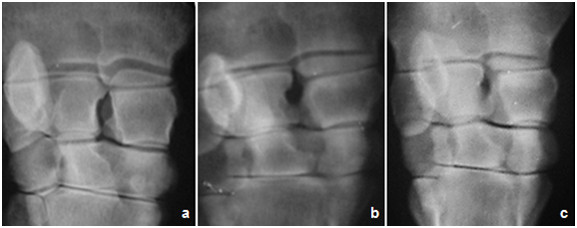
**Anterio-posterior radiographic image of the carpal joint, showing different arthritic changes in the untreated joints manifested by mild, moderate and severe narrowing of the joint spaces at (a) 1 month, (b) 2 month and (c) 6 month respectively, post injection of Amphotericin-B**.

**Figure 6:**
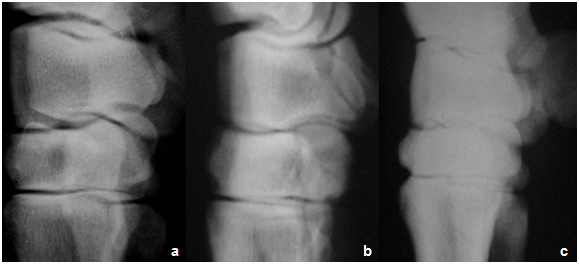
**Latro-medial radiographic image of the carpal joint, showing Different arthritic changes manifested by mild, moderate and severe narrowing of the joint spaces at (a) 1 month, (b) 2 month and (c) 6 month respectively, post injection of Amphotericin-B**.

**Figure 7:**
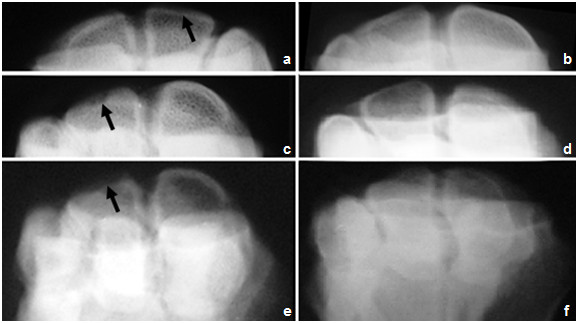
**Flexed radiographic image of the carpal joint of group-III, showing the cartilage at (a) 1 month, (b) 2 month and (c) 6 month post injection of Amphotericin-B **. Notice the thining of the articular cartilage compared to treated carpal joints -images on the right- at (d) 1 month, (e) 2 month and (f) 6 month post treatment with MSCs. Compare arrow with its contralateral.

Pre-injection of GFP-labeled MSCs: joints of all animals in **group-I **(3 weeks of induction) were less affected and the joint spaces were radiologically normal (score 0) whereas those of **group**-**II **and **group**-**III **(6 and 9 weeks post-induction respectively) showed narrowing of the joint spaces (score 2) with moderate degree of bone lysis in **group-III **(score 3). In the flexed view, thinning of the articular surface was noticeable. The thinning degree was higher in animals of **group-III **(score 4) than those of **group-I **(score 0) or **group-II **(score 2) (Figures 5, 6 and 7).

Post-injection of GFP-labeled MSCs: in **group-I**, there was no noticeable change in joint status in animals after 1 month of treatment. Joint were radiologically normal (score 0) and improvement degree did not differ much at 2 and 6 month follow up. In **group-II**, there was no noticeable change in joint status in animals after 1 month of treatment (score 2). Joint improvement was obvious after 2 months follow up (score 1). Improvement degree did not differ much at 6 month follow up (score 1). In **group-III**, no noticeable change in joint status was recorded in animals after 1 or 2 months of treatment (score 4). Mild joint improvement was seen after 6 month follow up (score 3). Radiological scoring of all animals was summarized in table 4.

**Table 4:** Mean value of the radiographic scoring of osteophytes formation and cartilage loss of different groups; before and at 1, 2, 6 months from the treatment

Assessment time	Group-I		Group-II		Group-III	
**Before Treatment**	0(2)		2(3)		4(4)	

	**R**	**L**	**R**	**L**	**R**	**L**

**1 month After Treatment**	0	0	2	3	4	4

**2 month After Treatment**	0	0	1	3	4	4

**6 month After Treatment**	0	0	1	2	3	4

**R**: right joint; **L**: left joint; **(Values between brackets) **indicate score of cartilage loss.

#### II-3-Synovial fluid analysis

**Figure 8:**
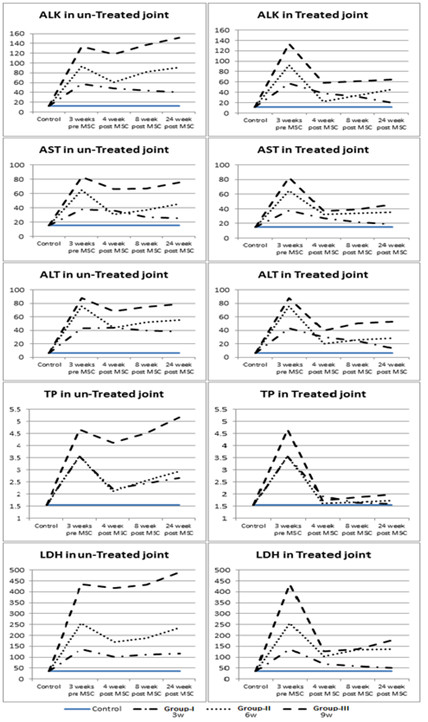
**Histograms showing physical, cytological and biochemical characteristics of synovial fluid analysis of the control and experimental groups**.

Physical, cytological and biochemical characteristics of synovial fluid analysis according to [30] of all animal models of OA are summarized in table (5 and 6) and Figure 8.

**Table 5:** Summary of the biochemical characteristics of synovial fluid analysis (Mean +/-SD)

	TP	AST	ALT	ALK	LDH
**Control**	1.56 ± 0.06	15.33 ± 2.76	6 ± 2	12 ± 2.0	36.5 ± 11.5

**3 weeks**	3.56 ± 0.04	38 ± 4.0	42.66 ± 6.6	57 ± 5.0	136.66 ± 8.6

**6 weeks**	3.58 ± 0.38	64.66 ± 4.4	75.33 ± 5.3	92.66 ± 4.6	256.66 ± 16.6

**9 weeks**	4.66 ± 0.4	82.66 ± 2.4	88 ± 4.0	133.33 ± 2.6	435.33 ± 20.0

*TP: total proteins; AST: aspartate aminotransferase; ALT: Alanine aminotransferase; ALK: alkaline phosphatase; and LDH: Lactic acid dehydrogenase*.

**Table 6:** Summary of Total proteins values in all 3 groups at different stages of the experiment

Total protein	Group-I	Group-II	Group-III
**Control**	1.56	1.56	1.56

**pre treatment**	3.56	3.58	4.66

**1 month post treatment**	1.91 (2.21)	1.61 (2.11)	1.73 (4.12)

**2 month post treatment**	1.65 (2.43)	1.66 (2.56)	1.88 (4.51)

**6month post treatment**	1.6 (2.66)	1.73 (2.93)	1.99 (5.16)

**(Values between brackets) **indicate values of non-cell treated animals; left joints.

#### II.4- Macroscopic appearance

**Figure 9:**
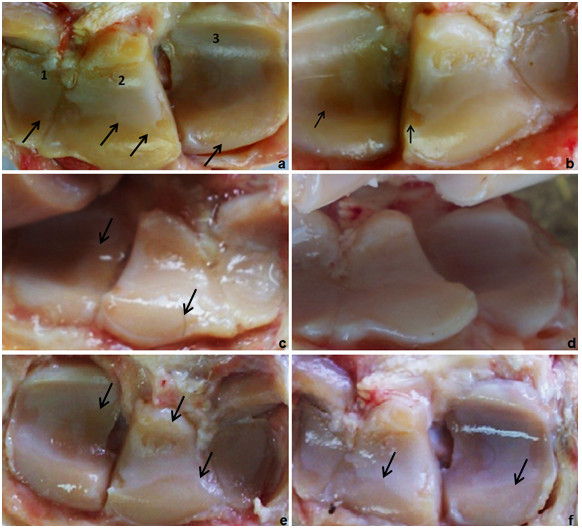
**(a) left antebrachiocarpal joint after 9 weeks of OA induction and 6 month of no treatment showing severe articular surface degeneration affecting the 1: ulnar carpal bone, 2: intermediate carpal bone and 3: radial carpal bone**. Notice the degenerative line marked by the arrow; (b) right antebrachiocarpal joint after 9 weeks of OA induction and 6 month of MSCs treatment showing improved articular surface. Notice the partial degenerative areas marked by the arrows; (c) left untreated joint of group-I after 2 month follow-up and (d) right treated joint; (e) untreated joint of group-II after 6 month follow-up (f) treated joints. Arrows indicates areas of degeneration. Compare left images (non-cell-treated) with the contralateral (cell-treated) ones.

Macroscopically, the articular surfaces showed different arthritic changes varied from slight discoloration to osteophytes formation. The magnitude of articular discoloration, erosions and articular surface roughness was higher in group-III than the other two groups (Figure 9a). The antebrachiocarpal joint after 2 months of OA induction and 6 month of no treatment showed severe articular surface degeneration affecting the carpal bones. In the cell-treated joints, noticeable rebuild up of the damaged cartilage was observed (Figure 9b). Likewise, in group-I the only noticeable changes were slight discoloration of the articular surface macroscopic where the normal bluish-white surface has become yellowish in color together with some capsulitis identified by petechial hemorrhagic spots on the joint capsule (Figure 9c). Slight differences could be noticed between the Cell-treated joints and none-cell-treated ones (Figure 9d).

In moderate cases of group-II, discoloration, slight erosions (Figure 9e) and slight roughness of the articular surface existed. Cell-treated joints showed evidences of better improvement than none-cell-treated ones (Figure 9f).

### II-5.Microscopic hisopathology

In order to evaluate the possible changes between the MSCs-treated and untreated groups, histological assessment of the articular surface of the radio-carpal joint from all animals was done using fluorescence microscopy analysis of the cell-treated joints together with regular and cartilage special dyes.

#### II-5.1.MSCs homing & florescence assessment

**Figure 10:**
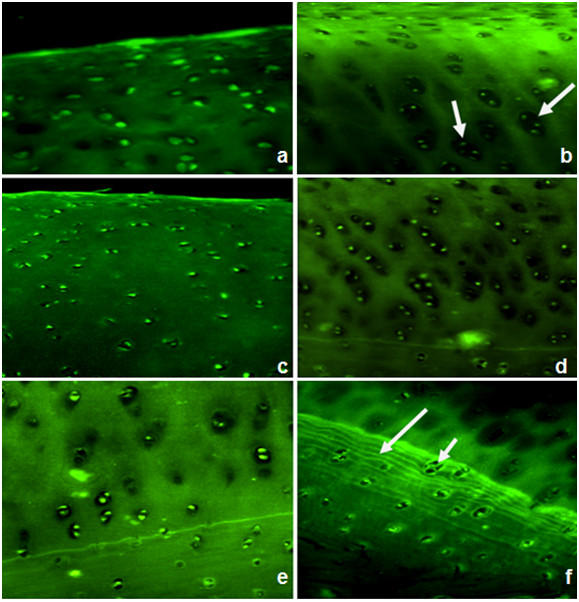
**Fluorescence microscopic analysis of the cell-treated joints showing GFP-positive cells detected at the surface and also in the center of regenerated tissue in all groups**. Group-I; (a) at 2 months and (b) at 6 month after injection of GFP-transduced MSCs. Group-II; show diffuse hypercellularity after 2 months (c) and clusters of chondrocytes after 6 months of injection (d). Group-III; show slight hypercellularity after 2 month (e) with clusters of chondrocytes (short arrow) and multiple tide marks (long arrow) after 6 months of injection (f).

Fluorescence microscopy of sections of the cell-treated joints of all groups indicated that the GFP-transduced implanted cells were integrated with in the articular cartilage. The cells were associated with the surface of the cartilage and, were also detected in the interior (Figure 10).

#### II.5.2.Hiastopathological assessment

Reduction of articular cartilage matrix staining, changes, osteophyte formation, and subchondral bone plate thickening were graded as described in table 7 according to [20] and results were as follows:

**Table 7:** Statistical significance of all treated groups at different follow up periods

Group/follow up period	Control Median &(Q1, Q3)*	Cases Median &(Q1, Q3)*	P- value
**Group-I: 2months**	10, (9, 10)	8, (7, 8)	0.043
**6 months**	16, (16, 18)	13, (12, 13)	0.043
**Group-II: 1 month**	10, (9, 10)	6, (5, 6)	0.043
**2 months**	14, (14, 15)	11, (10, 11)	0.043
**6 months**	18, (17, 18)	15, (14, 15)	0.043
**Group-III: 1 month**	15, (14, 15)	12, (11, 13)	0.046
**2 months**	17, (16, 17)	14, (14, 15)	0.043
**6 months**	19, (19, 19)	17, (17, 18)	0.034

*Q1 = 1^st ^quartile or 25^th ^percentile Q3 = 3^rd ^quartile or 75^th ^percentile

### Group-I

#### Post- injection termination after 1 month

Animals in this group died before the date of assessment and so were not histopathologically evaluated.

#### Post- injection termination after 2 months

**Figure 11:**
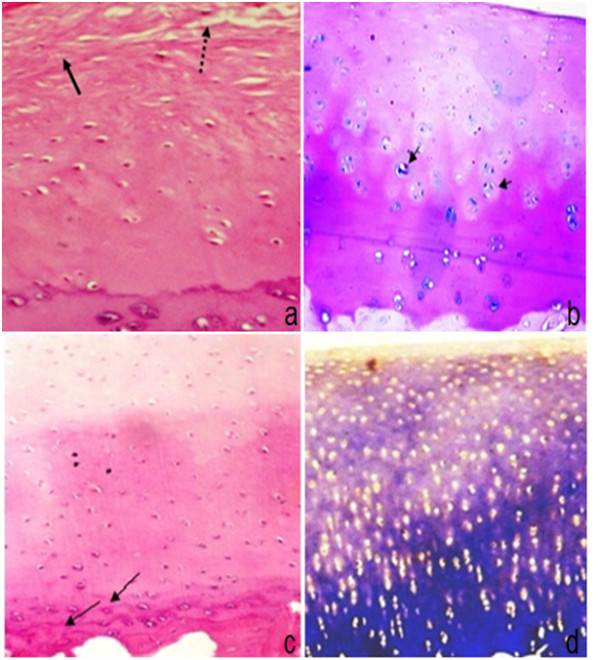
**Articular cartilage of group-I two months post injection: (a) control joint showing degenerative changes in the form of hypocellularity, fibrillation (arrow) & fissures (dotted arrows) (H&E 200X); (b) MSCs treated joint showing regenerative changes in articular cartilage including diffuse hypercellularity chondrocyte clones (arrows), regular surface and moderate decrease in matrix staining in superficial and middle zones (Alcian blue-PAS 100X)**. Articular cartilage of group-I six months post injection: (c): control joint showing duplicated tide marks (arrows) and moderate thickening of subchondral bone plate (H&E 100X); (d) MSCs treated joints showing regenerative changes in the form of moderate decrease of staining intensity of extracellular matrix with hypercllularity (MT 100X).

Obvious difference was seen between control and treated joints; Control joints showed fibrillation of 1/3 to 2/3 of articular cartilage, prominent duplicated tide mark, clusters of chondrocytes, calcification, and mild reduction of matrix staining. And mild subchondral bone plate thickening. Histopathologic scores were 9, 10, 10 (Figure 11a).

Treated joints: histologic scores for all parameters were closer to normal in cell-treated joints, and treatment had a significant effect on maintenance of the articular cartilage structure and subchondral bone plate thickening. Histopathologic scores were 6, 6, 5 (Figure 11b).

#### Post-injection termination after 6 months

##### Control group

There was hypocellularity, atrophic cells, erosions and focal areas of bone eburnation, moderate reduction of matrix staining, osteophyte formation of connective tissue with mild subchondral bone plate thickening (Figure 11c). Histopathologic scores were 16, 16, and 18 respectively.

On the other hand, treated joints showed diffuse hypercellularity, chondrocyte clusters, more calcification and less erosion (Figure 11d). Histopathologic scores were 11, 12, and 13 respectively.

### Group-II

#### Post- injection termination after 1 month

**Figure 12:**
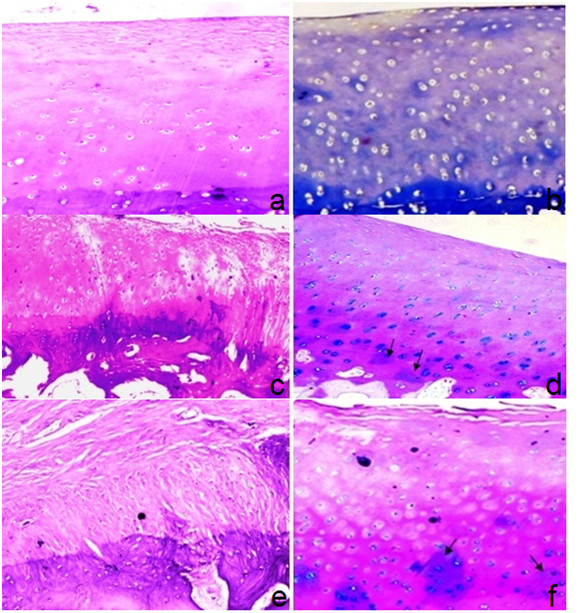
**Articular cartilage of group-II one month post injection: (a) control joint showing superficial fibrillation & clefts involving 1/3 with slight hypocellularity (H&E 100X); (b): MSCs-treated joint showing hypercellularity and marked decrease in staining with focal areas showing synthesis of extracellular matrix (arrows) (MT 100x)**. Articular cartilage of group-II two months post injection: (c) control joint showing irregular surface with superficial fibrillation & clefts involving up to 2/3 with slight hypercellularity and moderate subchondral bone thickening (H&E 100x); (d)MSCs, treated joint showing regenerative changes in articular cartilage with slight hypercellularity and increased matrix synthesis (arrows) in lower zone (Alcian blue-PAS 100x). Articular cartilage of group-II six months post injection: (e) control joint showing near total replacement of articular cartilage with fibrous tissue, loss of chondrocytes and marked subchondral bone thickening (H&E 100x); (f) MSCs-treated joint showing degenerative changes in the form of irregular surface, fissures and hypocellularity in superficial & middle zone and regenerative changes in lower zone denoted by increased matrix synthesis (arrows) (Alcian blue-PAS 100x).

The control joints show irregular surfaces; fissures, markedly reduced extracellular matrix, and osteophyticformation.Histopathologic scores were 9, 10, and 10 respectively (Figure 12a).

In the cell-treated joints, the degree of cartilage destruction, osteophyte formation, and subchondral sclerosis were all reduced compared with that in the control joints indicating that there was slow progression of the O.A. changes (Figure 12b). Histopathologic scores were 7, 8, and 8 respectively.

#### Post- injection termination after 2 months

The control joints treated with hyaluronic acid injection showed substantial fibrillation of the articular surface with loss of extracellular matrix, as well as large areas of osteophytic formation (Figure 12c). Histopathologic scores were 14, 14, and 15 respectively.

In the cell-treated joints, findings were better compared to this group with less severe arthritic changes. Histopathologic scores were 10, 11, and 11 respectively (Figure 12d).

#### Post-injection termination after 6 months

There were significant OA lesions in both the cell-treated and control joints. Histopathologic scores for control group were 17, 18, and 18 (Figure 12e) while for the cell-treated group were 14, 14, and 15 respectively (Figure 12f).

#### Group-III

**Figure 13:**
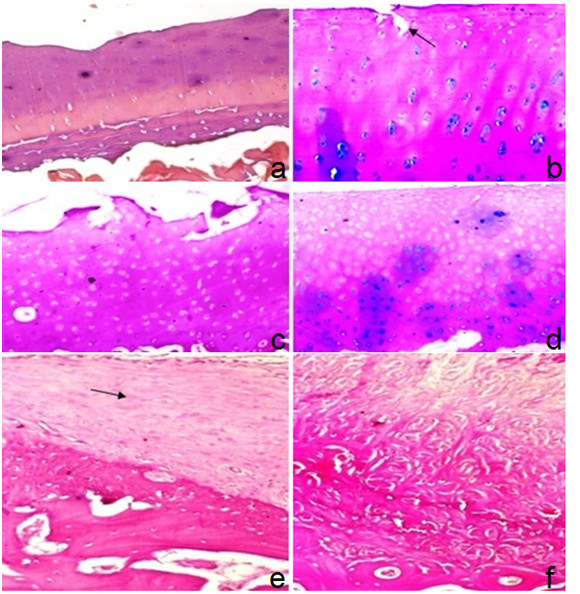
**Articular cartilage of group-III one month post injection: (a) control joint showing surface irregularity with hypocellularity and marked multiple tide marks (H&E 100x); (b) MSCs- treated joint showing degenerative changes with irregular surface, erosion (arrow) and hypocellularity in superficial zone and regenerative changes in middle zone & lower zone denoted by increased matrix synthesis, slight hypercellularity and chondrocytes clones (Alcian blue-PAS 100x)**. Articular cartilage of group-III two months post injection: (c) control joint showing surface erosion of articular cartilage with superficial loss of chondrocytes and moderate hypercellularity of rest of cartilage (H&E 100x); (d) MSCs-treated joint showing degenerative changes in the form of irregular surface, hypocellularity in superficial & middle zone and regenerative changes in lower zone denoted by hypercellularity& increased matrix synthesis (Alcian blue-PAS 100x). Articular cartilage of group-III six months post injection: (e) control joint showing articular cartilage destruction, pannus formation (arrow) and marked subchondral plate thickening (H&E 100x); (f) MSCs-treated joint showing articular cartilage destruction, fibrous tissue, scattered atrophic chondrocytes and marked subchondral plate thickening (H&E 100x).

Control cases in all subgroups showed marked OA changes in the form of erosions, pannus bone eburnation, osteophytic formation, loss of matrix staining, moderate to severe subchondral plate thickening (Figure 13a, c, and 13e). Histopathologic scores ranged from 15 to 19.

Cell-treated joints show nearly the same findings with slight difference or improvement of the articular cartilage status (Figure 13b, d and 13f). Histopathologic scores ranged from 11 to 18.

In all groups with different follow up periods there was significant difference between MSCs-treated cases and control cases (P- value < 0.05) (table 6).

## Discussion

Several tissue-engineering approaches have been used for the repair of joint lesions. Techniques that cause multipotent adult mesenchymal stem cells (MSCs) to differentiate into cells of the chondrogenic lineage have led to a variety of experimental strategies. In certain joint degenerative diseases such as osteoarthritis (OA), stem cells are depleted and have reduced proliferative capacity and reduced ability to differentiate [31]. The systemic or local delivery of stem cells to these individuals may therefore enhance repair or inhibit the progressive loss of joint tissue [20]. This study evaluated the effect of stem cell therapy suspended in hyaluronic acid for repair or delaying the progression of arthritic lesions that occur following joint injury and compared it to the use of hyaluronic acid alone.

Amphotericin-B was used in this work for induction of arthritis. Efficacy of Amphotericin-B in arthritis induction was reported in a previous work [14]. Chemical induction of OA when compared to surgical methods is an easy, rapid and less invasive technique [32]. Intra-articular injection of Amphotericin-B is an effective method to induce a synovitis/arthritis model and lameness in cattle. The histopathological degenerative changes that were obtained in this study by chemical induction was found to be comparable with the findings described [33] who used the surgical method for OA induction in the knee joint of rats.

Clinically, the immediate lameness after amphotericin B injection and joint swelling could be attributed to the initiation of an inflammatory process that caused synovitis and capsulitis. This can be due to the toxic effect of Amphotericin-B on the cartilage matrix as well as the synovial and capsular membranes with subsequent increase of synovial production [34]. Amphotericin-B has a direct toxic effect on chondrocyte resulting in the commencement of osteoarthritic changes in the injected joint [10]. In the study the lameness were more intense in group-III (score 5) than the other two groups. When treatment started, the degree of improvement depended mainly on the initial degree of severity of the condition, hence group-I showed better mending, (score 0 after 6 months of treatment), than the other two groups. Evidences of arthritic changes in the form of joint swelling and joint capsular distention were sighted, also radiographically, in all animals injected with Amphotericin-B in group-II and III. No visible radiographic signs of neither bone lysis nor osteophyte formation was seen in animals of group-I. Moderate and severe arthritic changes were observed in group-II group-III consequently. The severity of the alteration was in direct relationship to the induction period of arthritis similar to the results reported by (35, 8, and 10). Macroscopically, the different magnitudes of the articular damage could be insinuated as a result of the harmful effect of the injected Amphotericin-B. It is stated that Amphotericin-B causes lysosomal damage with liberation of its contents in the joint compartment. This observation resembles the tissue reaction in ponies using filipins (34, 8). As a result of the alteration in the articular surface nature, osteophyes starts to build-up cause the roughness in severe cases as seen in cases of group-III.

In this study, and agreed with other literatures, it was anticipated that established equine MSCs cultures would be negative for CD34, and positive for CD29 [36]. Cells were also characterized by their abilities to differentiate into chondrocytes and osteocytes. In this study, the ability of GFP transfected mesenchymal stem cells to be easily detected and its longevity enhanced our hypothesis for successful homing. Because the cells used in the present study were retrovirally transduced to express GFP, it is conceivable that expressed GFP or the vector used for the transduction may have affected the outcome. However, transduction of the cells did not affect their capacity to proliferate, and engraftment of the transduced cells in the articular cartilage occurred without evidence of an immune response at this site or elsewhere in the joint. The same conclusion was reported by [20].

Homing was proved in all injected specimens after 1, 2, and 6 months of follow up as GFP-labeled injected MSCs were detected in all examined articular cartilages. Some cells exhibited a chondrocyte-like phenotype (rounded form; surrounded by a lacuna) indicating differentiation of injected MSCs while in other areas cells remained spindle-like (mesenchymal). Thus we can propose that the local environment of the homing site can induce a chondrogenic phenotype in undifferentiated MSCs [37].

Previous study showed that implanted PKH 26-labeled MSCs were identified in the newly formed bony trabeculae in specimens at 2 and 4 months after implantation. These results offer a potential approach to meet clinical requirements in the treatment of infected bone defects [38].

Another previous study was compared the *in vivo *chondrogenic potential of synovial MSCs, bone marrow MSCs, adipose MSCs, and muscle MSCs by transplanting them into cartilage defects in rabbits. Synovial MSCs and bone marrow MSCs had much more chondrogenic potential than adipose MSCs and muscle MSCs [39].

In this study, MSCs were suspended in sodium hyaluronate before injection and control joints were injected by sodium hyaluronate only. As regards the control joints, the histological findings reflected the degenerative nature of this model as presence of surface cartilage irregularities and fibrillation, edema, hypocellularity alternating with hypercellular & proliferative areas and decrease of the concentration of proteoglycans characterized by the reduction of staining intensity. Such changes are observed in the DJD and were also described by [40,41].

The absence of obvious beneficial effect of the sodium hyaluronate alone on the structure of the degenerate articular cartilage was evident by histological scores. Comparable results were described by [42-44].

The presence of proliferation of chondrocytes may be explained as the injury to the structure of the cartilage produced death of the chondrocytes and initiated a repair response, resulting in chondrocyte proliferation. Sandell and Aigner in 2001 [45] have described similar changes of the articular cartilage to counteract losses occurred during DJD, by the increase of chondrocyte proliferative activity.

Some authors [46,47] reported that hyaluronan contributes to the granulation phase of wound healing and stimulates the migration and mitosis of mesenchymal and epithelial cells. So, MSC-based repair in the presence of hyaluronan may therefore accelerate and amplify the natural repair process of recruiting these cells to the site of tissue repair or regeneration.

The involvement of injected MSC in the development of appreciable neocartilagenous tissue in treated joints was associated with protection against more severe degenerative changes when compared with control joints. However, the continuing degradation of treated joints that occurred at prolonged time points indicate the need for some augmentation of natural repair by MSCs. This is evidenced by the histopathologic scoring of different groups where the score of MSCs-treated cases in group-I after 2 months follow up were 7, 8, and 8 and in control joints were 9, 10, and 10 while after 6 months follow up the scores were 12, 13, 13 and in control joints were 16, 16, and 18 respectively. Other evidence could be noticed by comparison of the previous results with that of group-III after 6 months follow up period where score of MSCs-treated cases were 17, 17, 18 and in control joints were 19 in all cases denoting that Cell-treated joints show slight difference or improvement of the articular cartilage status.

It was noticed that the mean histopathological scores of the stem cell treated group was smaller than the control group mean score in the 3 studied groups. It reflected the presence of less conspicuous degenerative injuries, assuming that mesenchymal stem cells stimulated the reparative process or delayed the disease evolution. The difference among the treated groups and control ones was significant (P < 0.05). This beneficial effect on the degenerate cartilage was also described by [20].

The best effect of MSCs on different degrees of arthritis was not clearly concluded from our results except that the effect in group-III was minimal. Also, the grading scores in the treated cases in group-II were greater than those of group-I. It is likely that the cumulative effect of the abnormal load imposed as a result of the severed cartilage resulted in progressive cartilage damage that was not completely prevented by the repair process. So we concluded that the earlier the injection the better the effect. However, these results should be applied on large number of animals for better evaluation.

## Conclusion

Augmented therapeutic effect was proved with intra-articular injection of stem cells suspended in hyaluronic acid than the injection of hyaluronic acid alone following injury of the joint. This injection offers repair of affected joint and reduction or delay in the progression to OA. Earlier injection of MSCs is more beneficial.

We are dealing with progressive degenerative disease and our results show that the animal score deteriorate by time. MSC only delay this deterioration but doesn't improve it totally so we may need repeated injections to reach better results. Although this study had a follow up period of 6 months, longer term follow up is mandatory to study the permanency of the effect and fate of injected cells.

## Abbreviation

MSC: Mesenchymal stem cell; GFP: Green fluroscent protein; I.A.: Intra-aticularily; O.A.: Osteoarthritis; DPBS: Dulbecco's phosphate-buffered saline; BMP-2: Bone morphogenetic protein-2; TGF ß3: Transforming growth factor ß3; APA: American Psychological Association; MT: Masson's trichrome; H&E: Haematoxylene and eosin; SPSS: Statistical package for social science.

## Declaration of competing interests

The authors declare that they have no competing interests.

## Authors' contributions

AM: participated in the design of the study, drafting the article after collecting the data done by the other authors and revising it critically for important intellectual content. OE: participated in the design of the study, carried out the acquisition of the MSCs from the bone marrow, and creation of the defects and clinical assessment of the animal during the study period. AS: provision of the study materials, technical and logistic support and carried out intra-articular injection of the labeled MSCs. DS and LA: carried out the isolation and characterization of the mesenchymal stem cells, and participated in labeling of the cell. AM: carried out the preparation of the specimens, confirming the homing of the cells and interpretation of the effect of the injection on quality of the repair tissue. All authors read and approved the final manuscript.

## Pre-publication history

The pre-publication history for this paper can be accessed here:

http://www.biomedcentral.com/1471-2474/12/259/prepub

## Supplementary Material

Additional file 1 **All groups of animals' data sheet format of the present work of the study**. Timetable sheet format provided showing aspiration of the synovial fluid and at the same procedure; each animals received its designated autologous MSCs IA injection coupled with hyaluronic acid on its right carpal joint, while the left carpal joint was injected with hyaluronic acid only. Click here for file

## References

[B1] Caplan AI Dennis JE Mesenchymal stem cells as trophic mediators Journal of Cellular Biochemistry 2006 98 5 1076 1084 10.1002/jcb.20886 16619257

[B2] Sordi V Mesenchymal Stem Cell Homing Capacity Transplantation 2009 87 9S S42 S45 19424004 10.1097/TP.0b013e3181a28533

[B3] Barry FP Biology and clinical applications of mesenchymal stem cells Birth Defects Res C Embryo Today 2003 69 3 250 6 10.1002/bdrc.10021 14671778

[B4] Nöth U Steinert AF Tuan RS Technology Insight: adult mesenchymal stem cells for osteoarthritis therapy: Delivery modes for Mesenchymal stem cells Nature Clinical Practice Rheumatology 2008 4 371 380 18477997 10.1038/ncprheum0816

[B5] Aigner T Soder S Gebhard P McAlinden A Haag J Mechanisms of disease: role of chondrocytes in the patho- genesis of osteoarthritis--structure, chaos and senescence Nat Clin Pract Rheumatol 2007 3 391 399 10.1038/ncprheum0534 17599073

[B6] Di Cesare PE Abramson SB Samuels J Firestein GS, Firestein et al Pathogenesis of Osteoarthritis 2008 Kelley's Textbook of Rheumatology, 8E, (Chapter 89), Philadelphia

[B7] Ameye LG Young MF Animal Models of Osteoarthritis: Lessons Learned While Seeking the 'Holy Grail' Curr Opin Rheumatol 2006 18 5 537 547 10.1097/01.bor.0000240369.39713.af 16896297

[B8] Bowman KF Purohit RC Ganjam VK Pechman RD Jr Vaughan JT Thermographic evaluation of corticosteroid efficacy in Amphotericin-B -induced arthritis in ponies Am J Vet Res 1983 44 51 56 6824225

[B9] Crawford WH Houge JC Neirby DT Di Mino A Di Mino AA Pulsed radio frequency therapy of experimentally induced arthritis in ponies Can J Vet Res 1991 55 76 85 1884288 PMC1263418

[B10] Fahmy AS Hegazy AA Abdelhamied MA Shamaa AA Schimke E Clinical, Biochemical and Histopathological studies on arthritis in equine Vet Med J Giza 1994 42 1 305 320

[B11] Hegazy AA Fahmy LS Fahmy AS Abdelhamied MA Shamaa AA and Schimke E Evaluation of the effects of intra-articular injection of dimethylsulfoxide on chemically induced arthritis in equines Vet Med J Giza 1994 42 221 243

[B12] Suominen MM Tulamo RM Puupponen LM Sankari SM Effects of intra-articular injections of bufexamac suspension on Amphotericin-B -induced aseptic arthritis in horses Am J Vet Res 1999 60 12 1467 73 10622153

[B13] Marttinen PH Raulo SM Suominen MM Tulamo RM Changes in MMP-2 and -9 activity and MMP-8 reac- tivity after Amphotericin-B induced synovitis and treatment with bufexamac J Vet Med 2006 53 311 318 10.1111/j.1439-0442.2006.00837.x 16901276

[B14] Shamaa A Mokbel A El-Tookhy O Mostafa A The efficiency of Intra-articular injection of Amphotericin-B in inducing arthritis in experimental Equine model Accepted at 11th Scientific conference (3rd International) 15-18, May 2011, Faculty of Veterinary Medicine, Cairo University

[B15] Jiang W Ma A Wang T Han K Liu Y Zhang Y Dong A Du Y Huang X Wang J Lei X Zheng X Homing and differentiation of mesenchymal stem cells delivered intravenously to ischemic myocardium in vivo: a time-series study European Journal of Physiology 2006 453 1 43 52 10.1007/s00424-006-0117-y 16915405

[B16] Kollar K Cook MM Atkinson K Brooke G Molecular Mechanisms Involved in Mesenchymal Stem Cell Mi- gration to the Site of Acute Myocardial Infarction International Journal of Cell Biology 2009 2009 904682 pages 8 20130773 10.1155/2009/904682 PMC2809335

[B17] Syková E Jendelová P Urdzíková L Lesný P Hejcl A Bone marrow stem cells and polymer hydrogels-two strategies for spinal cord injury repair Cell Mol Neurobiol 2009 26 7-8 1113 29 10.1007/s10571-006-9007-2 PMC11520705 16633897

[B18] Hu SL Luo HS Li JT Xia YZ Li L Zhang LJ Meng H Cui GY Chen Z WuN Lin JK Zhu G Feng H Functional recovery in acute traumatic spinal cord injury after transplantation of human umbilical cord mesenchymal stem cells Critical Care Medicine 2010 38 11 2181 2189 10.1097/CCM.0b013e3181f17c0e 20711072

[B19] Miller RH Bai L Lennon DP Caplan AI The Potential of Mesenchymal Stem Cells for Neural Repair Discovery Medicine 2010 9 46 236 242 20350491

[B20] Murphy MJ Fink DJ Hunziker EB Barry FP Stem cell therapy in a Caprine model of osteoarthritis Arthritis & Rheumatism 2003 48 12 3464 3474 10.1002/art.11365 14673997

[B21] El-Tookhy O AbouElkheir W Mokbel A Osman A Intra-articular Injection of Autologous Mesenchymal Stem Cells in Experimental Chondral Defects in Dogs Egypt Rheumatologist 2008 30 2 1 10 21385540

[B22] American Psychological Association Guidelines for Ethical Conduct in the Care and Use of Animals 1996 10.1901/jeab.1986.45-127 PMC1348222 16812447

[B23] Anon J Guide for Veterinary Service and Judging of Equestrian Events 1991 4 Lexington KY: Am Assoc Equine Practnr

[B24] Martin A Sandra O Mandi J Daniel B Olga B Jill R Rustin M Jeffrey M Comparison of Chondrogenic Potential in Equine Mesenchymal Stromal Cells Derived from Adipose Tissue and Bone Marrow Vet Surg 2008 37 8 713 724 10.1111/j.1532-950X.2008.00462.x 19121166 PMC2746327

[B25] Radcliffe CH Flaminio MJ Fortier LA Temporal Analysis of Equine Bone Marrow Aspirate During Establishment of Putative Mesenchymal Progenitor Cell Populations 2010 19 2 Stem cells and Development 10.1089/scd.2009.0091 PMC3138180 19604071

[B26] Zhi-Yong Z Swee-Hin T Mark S Jan T Nicholas M Mahesh A Jeery C Superior Osteogenic Capacity for Bone Tissue Engineering of Fetal Compared with Perinatal and Adult Mesenchymal Stem Cells Stem cells 2009 27 126 137 10.1634/stemcells.2008-0456 18832592

[B27] Niki H Hosokawa S Nagaike K Tagawa T A new immunofluorostaining method using red fluorescence of PerCP on formalin-fixed paraffin-embedded tissues J Immunol Methods 2004 293 1-2 143 51 10.1016/j.jim.2004.07.009 15541284

[B28] Maurisse R De Semir D Emamekhoo H Bedayat B Abdolmohammadi A Parsi H Gruenert D Comparative transfection of DNA into primary and transformed mammalian cells from different lineages BMC Biotechnology 2010 10 9 10.1186/1472-6750-10-9 PMC2830169 20144189

[B29] Eronen I Videman T Friman C Michelsson JE Glycosaminoglycan metabolism in experimental osteoarthrosis caused by immobilization ActaOrthop Scand 1978 49 4 329 34 10.3109/17453677809050083 696272

[B30] Carlson CS Guilak F Vail TP Gardin JF Kraus VB Synovial fluid biomarker levels predict articular cartilage damage following complete medial meniscectomy in the canine knee J Orthop Res 2002 20 92 100 10.1016/S0736-0266(01)00066-3 11853096

[B31] Murphy JM Dixon K Beck S Fabian DF Feldman A Barry FP Reduced chondrogenic and adipogenic activity of mesenchymal stem cells from patients with advanced osteoarthritis Arthritis Rheum 2002 46 704 13 10.1002/art.10118 11920406

[B32] Kotschwar JL Coetzee JF Anderson DE Gehring R KuKanich B Apley MD Analgesic efficacy of sodium salicylate in an Amphotericin-B -induced bovine synovitis-arthritis model Dairy Sci 2009 92 3731 3743 10.3168/jds.2009-2058 19620655

[B33] Appleton CTG McErlain DD Pitelka V Schwartz N Bernier SM Henry JL Holdsworth DW Beier F Forced mobilization accelerates pathogenesis: characterization of a preclinical surgical model of osteoarthritis Arthritis Res Ther 2008 10 407 10.1186/ar2513 PMC1860072 17284317

[B34] Ayotte R Laurin CA Pathogenesis of joint effusions: Anexperimental study Can Med A 1969 100 242 250 PMC1945570 5763237

[B35] McIlwraith CW Fessler JF Blevins WE Page EH Rebar AH Van Sickle DC Coppoc GL Experimentally induced arthritis of the equine carpus: clinical determinations Am J Vet Res 1979 40 1 11 19 453671

[B36] Dominici M Le Blanc K Mueller I Slaper-Cortenbach I Marini F Krause D Deans R Keating A Prockop D Horwitz E Minimal criteria for defining multipotent mesenchymal stromal cells. The international society for cellular therapy position statement Cytotherapy 2006 8 315 317 10.1080/14653240600855905 16923606

[B37] Tatebe M Nakamura R Kagami H Okada K Ueda M Differentiation of transplanted mesenchymal stem cells in a large osteochondral defect in rabbit Cytotherapy 2005 7 6 520 530 10.1080/14653240500361350 16306014

[B38] Ueng SW Yuan LJ Lin SS Liu SJ Chan EC Chen KT Lee MS In vitro and in vivo analysis of a biodegradable poly (lactide-co-glycolide) copolymer capsule and collagen composite system for antibiotics and bone cells delivery J Trauma Jun 2011 70 6 1503 9 10.1097/TA.0b013e3181edb873 21336203

[B39] Koga H Shimaya M Muneta T Nimura A Morito T Hayashi M Suzuki S Ju YJ Mochizuki T Sekiya I Local adherent technique for transplanting mesenchymal stem cells as a potential treatment of cartilage defect Arthritis Res Ther 2008 10 4 R84. 18664254 10.1186/ar2460 PMC2575632

[B40] Mankin HJ Dorfman H Lipiello N Zarins A Biochemical and metabolic abnormalities in articular cartilage from osteo-arthritic human hips II: Correlation of morphology with biochemical and metabolic data J Bone Joint Surg Am 1971 53-A 523 537 5580011

[B41] Melo EG Gomes MG Nunes VA Rezende CMF Effects of chondroitin sulfate and sodium hyaluronate on chondrocytes and extracellularmatrix of articular cartilage in dogs with degenerative joint disease Arq Bras Med Vet Zootec 2008 60 1 93 102 10.1590/S0102-09352008000100014

[B42] Smith GN Myers SL Brandt KD Mickler EA Effect of intraarticular hyaluronan injection in experimental canine osteoarthritis Arth Rheum 1998 41 976 985 10.1002/1529-0131(199806)41:6<976::AID-ART4>3.0.CO;2-R 9627007

[B43] Guidolin DD Ronchetti IP Lini E Guerra D Frizziero L Morphological analysis of articular biopsies from randomized, clinical study comparing the effects of 500-730 kDa sodium hyaluronate (Hyalgan^®^) and methylpredinisolone acetate on primary osteoarthritis of the knee Osteoarth Cart 2001 9 371 381 10.1053/joca.2000.0398 11399102

[B44] Barbucci R Lamponi S Borzachiello A Ambrosio L Fini M Torricelli P Giardino R Hyaluronic acid hydrogel in the treatment of osteoarthritis. Biomaterials 2002 23 4503 4513 10.1016/s0142-9612(02)00194-1 12322970

[B45] Sandell LJ Aigner T Articular cartilage and changes in arthritis An introduction: cell biology of osteoarthritis Arthritis Res 2001 3 107 113 10.1186/ar148 11178118 PMC128887

[B46] McCarty MF Glucosamine for wound healing. Med Hypotheses 1996 47 273 275 10.1016/s0306-9877(96)90066-3 8910875

[B47] Chen WY Abatangelo G Functions of hyaluronan in wound repair Wound Repair 1999 7 79 89 10.1046/j.1524-475X.1999.00079.x 10231509

