# Supplementary figures and images for: Homing and reparative effect of intra-articular injection of autologus mesenchymal stem cells in osteoarthritic animal model
Source: BMC Musculoskelet Disord. 2011 Nov 15;12:259. doi: 10.1186/1471-2474-12-259 (PMC3232438; doi:10.1186/1471-2474-12-259)

**Additional file 1**


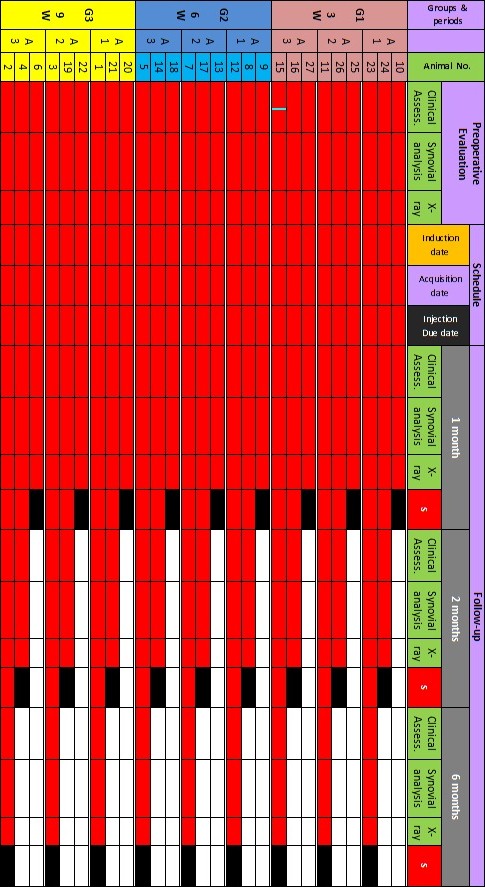


Data Sheet used in this work.

Supplement: Additional file 1 — All groups of animals' data sheet format of the present work of the study. Timetable sheet format provided showing aspiration of the synovial fluid and at the same procedure; each animals received its designated autologous MSCs IA injection coupled with hyaluronic acid on its right carpal joint, while the left carpal joint was injected with hyaluronic acid only. [file 1471-2474-12-259-S1.DOC]
